# Increased Incidence of Tumors With the *IKBKAP* Gene Mutation? A Case Report and Review of the Literature

**DOI:** 10.4021/wjon278w

**Published:** 2011-02-26

**Authors:** Marianna Shvartsbeyn, Amy Rapkiewicz, Felicia Axelrod, Horacio Kaufmann

**Affiliations:** a Department of Pathology, New York University, New York, USA; b Department of Pediatrics, New York University, New York, USA; c Department of Neurology, New York University, New York, USA

**Keywords:** Familial dysautonomia, IKBKAP, Tumorgenesis

## Abstract

An increased incidence of neoplasia was recently reported in patients with familial dysautonomia. This suggests that, in addition to its role in neuronal development, the *IKBKAP* gene may also influence DNA repair. Here we report the case of a 28-year-old male with familial dysautonomia who was found to have neoplastic lesions detected post mortem as incidental findings. This case indicates that the prevalence of tumorgenesis within this population may be underestimated.

## Introduction

Familial dysautonomia, also known as Riley-Day syndrome and hereditary sensory and autonomic neuropathy type III, is a rare autosomal recessive disorder. Over 99.5% of cases are homozygous for a point mutation in the 5’ splice site of the *IKBKAP* gene located on the long arm of chromosome 9 (9q31), which encodes IkB kinase complex associated protein (IKAP) [1, 2]. The carrier rate among Askenazi Jews is approximately 1 in 27. The disorder is characterized by sensory and autonomic dysfunction as a result of an abnormality in the development in the sensory and autonomic neuronal pathways [3].

The protein IKAP is predominantly present in the cytoplasm where it interacts with other proteins involved in cellular migration, adhesion and actin cytoskeleton organization [4]. The *IKBKAP* gene sequence bears a striking similarity to the *ELP1* gene, which encodes a subunit of a complex that binds to RNA polymerase II and is required for the activation and transcriptional elongation of a large number of genes [5]. Similar to ELP1, IKAP may be a part of the RNA polymerase II elongation complex and might play a role in expression of the genes involved in neural migration, differentiation and vascular development in utero [4, 6-9]. Interestingly, in vitro experiments showed that fibroblasts from familial dysautonomia patients had increased susceptibility to DNA damage after exposure to N-methyl-N’-nitro-N-nitrosoguanidine [10]. This finding suggests an inability to effectively repair damaged DNA and may explain the increased incidence of tumorgenesis in these patients.

## Case Report

The decedent was a 28-year-old Ashkenazi Jewish man diagnosed with familial dysautonomia by genetic testing. At 6 weeks of age he presented with hypothermia and severe feeding problems. He had alacrima and other characteristic features of familial dysautonomia including unstable blood pressure and gastrointestinal dysfunction with oral incoordination and gastroesophageal reflux. He underwent a Nissen fundoplication and gastrostomy at age 2, suffered three episodes of intestinal obstruction, requiring bowel resection, and multiple bouts of pancreatitis and cholecystitis, persistent esophageal dysmotility leading to multiple episodes of aspiration pneumonia. Cinesophagram demonstrated marked esophageal dilation with poor peristaltic wave and narrowed spastic esophagogastric junction. He had kyphoscoliosis and underwent spine fusion at the age of ten; he also had equinovarus deformity and osteoporosis with multiple bone fractures. He was classified as learning disabled. He suffered several seizures associated with hyponatremia and hypoxia. Because of obstructive sleep apnea and nocturnal oxygen desaturations and hypercapnia that persisted after tonsillectomy and adenoidectomy, non-invasive positive pressure respiration was recommended but refused. In the months preceding his death, his condition was stable and there was no obvious change in his status on the evening that preceded his unexpected death during sleep.

The salient findings at autopsy were the esophagus distended with partially undigested food and evidence of aspiration pneumonia with associated foreign body giant cell reaction on the lung sections. Taken together in a context of swallowing difficulty and esophageal dysmotility, these findings were consistent with aspiration as a cause of death.

**Figure 1:**
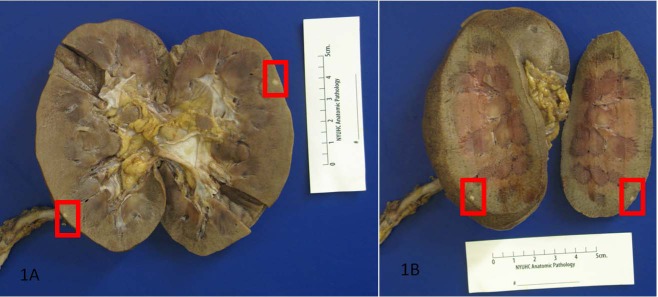
Autopsy revealed multiple well-circumscribed unencapsulated, pale yellow tumors in the subcapsular portions of both kidneys.

**Figure 2:**
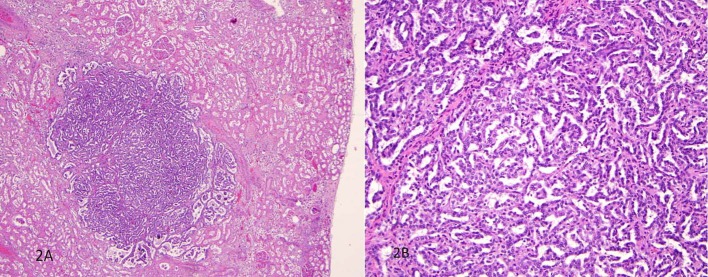
H&E sections revealed densely packed papillae or tubules lined by small, regular, cuboidal cells with rounded, uniform nuclei that lacked cytologic anaplasia and mitoses.

The unexpected findings were multiple well-circumscribed unencapsulated, pale yellow tumors in the subcapsular portions of both kidneys ranging from 0.2 to 0.4 cm in greatest dimension (Fig. 1). H&E sections revealed densely packed papillae or tubules lined by small, regular, cuboidal cells with rounded, uniform nuclei that lacked cytologic anaplasia and mitoses, making a diagnosis of tubulopapillary adenomas (Fig. 2).

## Discussion

The mechanism of tumorgenesis in familial dysautonomia is unknown. A recent retrospective review indicates that two percent of patients with familial dysautonomia had a neoplasm [11]. All were detected prior to death between the ages of 7 to 49, which suggests that mutations in the *IKBKAP* gene may be predisposed to neoplasia at an early age. Therefore, considering that most of these patients succumb to the complications of their disease at a young age and that religious Jews often refuse to allow autopsy, it is likely that other patients with familial dysautonomia, like our own case, may also have died with unrecognized tumors which would increase the incidence of neoplasias even further. Notably, the father of this patient passed away at a young age of unknown cancer. It may be of significance since carriers are known to have reduced levels of the *IKBKAP* gene. In one study, a strong correlation was found between the *IKBKAP* mRNA levels of the 22 familial dysautonomia patients and their parents [12], suggesting that the efficiency of the splicing is controlled by genetic mechanisms.

In our case, we found multiple bilateral tubulopapillary adenomas, which are low-grade papillary lesions with striking histological and genetic similarities to papillary carcinomas and are distinguished from the latter on the basis of size under 0.5 cm [13]. These small renal cortical epithelial neoplasms may be found incidentally. However, bilateral multifocal lesions are uncommon, unless they develop as a part of von Hippel-Lindau syndrome [14]. Although the previous review of neoplasms in familial dysautonomia included some low-grade neoplastic lesions [11], with time these proliferative lesions may have evolved into malignancy if the decedents had a full life expectancy.

The relationship between our findings and the *IKBKAP* gene mutations is unclear. Cytogenetic and molecular techniques demonstrated balanced translocations involving chromosome 9p and 9q (q34) in both clear cell and papillary renal cell carcinoma, the malignant counterparts of the adenomas [15, 16]. Perhaps, the proximity to the causative gene may play a role in this association. Alternatively, by virtue of affecting the critical part of the transcriptional machinery, *IKBKAP* gene expression may be involved in crucial signaling cascades affecting expression of multiple secondary genes, the effects of which are yet to be discovered. Finally, defective repair of damaged DNA may provide yet another mechanism for accelerated tumorgenesis in patients with familial dysautonomia.
